# Interleukin-1 Blockade With Rilonacept in Recurrent Pericarditis: Tapering and Discontinuation in Real-World Practice

**DOI:** 10.1016/j.jacadv.2026.102774

**Published:** 2026-04-24

**Authors:** Michele Marchetta, Georgia Thomas, Michele Golino, Jamey A. Cutts, Jonathan A. Pan, Rocio I. Lopez, Reid Clark, Sophia Bigio, Marco Giuseppe Del Buono, Antonio Abbate

**Affiliations:** a Robert M. Berne Cardiovascular Research Center, Division of Cardiology, University of Virginia, Charlottesville, Virginia, USA; b Department of Cardiovascular Medicine, Fondazione Policlinico Universitario A. Gemelli IRCCS, Rome, Italy; c Department of Internal Medicine and Pauley Heart Center, Virginia Commonwealth University, Richmond, Virginia, USA

**Keywords:** recurrent pericarditis, rilonacept, tapering

**Figure:**
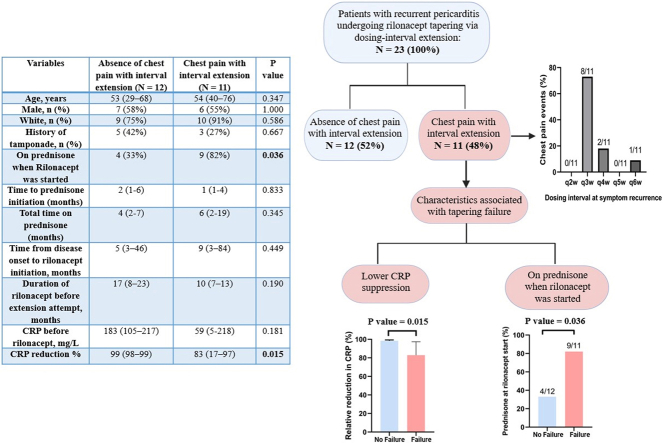

**What is the clinical question being addressed?**

Is rilonacept tapering via dosing-interval extension feasible in recurrent pericarditis, and which factors are associated with failure?

**What is the main finding?**

The tapering was feasible but often limited by recurrent chest pain; prednisone at initiation and less CRP suppression were associated with tapering failure.

Recurrent pericarditis (RP) affects 20 to 30% of patients after a first episode of pericarditis and can lead to a relapsing course.^1^ RP is now recognized as an interleukin (IL)-1 driven inflammatory disease. In the Rilonacept inHibition of interleukin-1 Alpha and beta for recurrent Pericarditis: a pivotal Symptomatology and Outcomes stuDY (RHAPSODY), rilonacept, a soluble IL-1 decoy receptor, demonstrated rapid symptom relief, C-reactive protein (CRP) normalization, and fewer recurrences, supporting its use in colchicine-resistant RP and reducing prednisone dependence.^1^^,^^2^ In RHAPSODY, recurrences were rare on therapy but common after discontinuation, with ∼75% of patients relapsing within 9 to 12 weeks.^2^ Although generally well tolerated, prolonged rilonacept therapy raises practical concerns, including high cost, injection-site reactions, and potentially blunted systemic signs of infection due to IL-1 blockade. The optimal treatment duration remains uncertain, and discontinuation in routine practice is often limited by concerns for recurrent symptoms. Saraswati et al reported higher recurrence rates after abrupt discontinuation rather than after gradual tapering, with post-discontinuation colchicine potentially reducing relapse risk.^3^ These data support a step-down approach and highlight the need for prospective studies to standardize tapering and long-term management.

## Methods

We herein report our experience with the use of rilonacept in patients with RP whose pericarditis was not secondary to a systemic immune-mediated disease. Our primary objective was to describe the success rate of a stepwise rilonacept tapering and to explore whether CRP suppression and prednisone exposure were associated with tapering failure, defined as recurrence of chest pain during dosing interval extension. CRP suppression was defined as the percentage decrease from baseline CRP to the lowest observed on-treatment CRP value during the first year of therapy. Patients met the 2025 American College of Cardiology Expert Consensus Statement criteria for RP.^1^ All patients had previously received first-line medical therapy when not contraindicated. Rilonacept was administered as a 320 mg loading dose followed by 160 mg subcutaneously once weekly. Tapering followed a stepwise interval extension protocol: rilonacept every 2 weeks for 4 weeks, then every 3 weeks for 6 weeks, with further extension to every 4, 5, and 6 weeks before discontinuation if no chest pain occurred before the next scheduled dose at each step. In the event of chest pain, CRP was measured, and the dosing interval was shortened by one step or returned to weekly dosing for severe symptoms or patient preference. A flare required chest pain plus objective inflammation (CRP >10 mg/L and/or imaging evidence of active pericardial inflammation by magnetic resonance imaging); chest pain without biochemical or imaging confirmation was classified as a nonflare event. Colchicine was continued throughout the tapering protocol and after rilonacept discontinuation. Continuous variables are reported as median (IQR), and categorical variables as absolute numbers (percentages). Continuous variables were compared between groups using the Mann-Whitney *U* test, and categorical variables were compared using Fisher exact test. Two-sided *P* value <0.05 was considered statistically significant. Analyses were performed with SPSS version 27. The University of Virginia Institutional Review Board determined the study to be exempt.

## Results

We included 23 patients, 13 (57%) male, age 54 (38-70) with viral or idiopathic pericarditis (13, 56%), post-cardiac injury (5, 22%), or post-vaccine (5, 22%). Over a total follow-up of 25 [21–40] months, all patients underwent rilonacept interval extension following a median of 12 [7–19] months of therapy. During interval extension, 11 (48%) developed chest pain and reverted to a shorter dosing interval after 2 [1–4] months, of which 8 (73%) had symptoms during transition from dosing every 2 weeks to every 3 weeks. Only 1 of 11 (9%) developed a true clinical flare with CRP elevation. Of these, 6 (55%) attempted a second taper; 2 (33%) subsequently discontinued after an additional 6 and 11 months, while 4 (66%) remain in a second taper attempt with 8 [3–14] months of follow-up. Among the 12 patients who did not report chest pain during tapering, 4 (33%) discontinued rilonacept at first tapering attempt, whereas the remaining 8 (67%) were maintained on extended dosing intervals for a median of 5 [3–6] months. At last follow-up, 17 of 23 (74%) remained on a tapered regimen: 5 (30%) had returned to weekly dosing, 6 (35%) every 2 weeks, and 6 (35%) every 3 weeks or longer.

Overall, rilonacept was discontinued in 6 (26%) patients who completed the tapering process, after 20 [18–24] months. Over 10 [4–12] months of follow-up after discontinuation, 2 (33%) patients relapsed at 8 and 11 weeks and restarted rilonacept, while 4 (67%) remained off rilonacept on colchicine monotherapy. Symptoms responded to rilonacept resumption in all cases.

**Figure 1:**
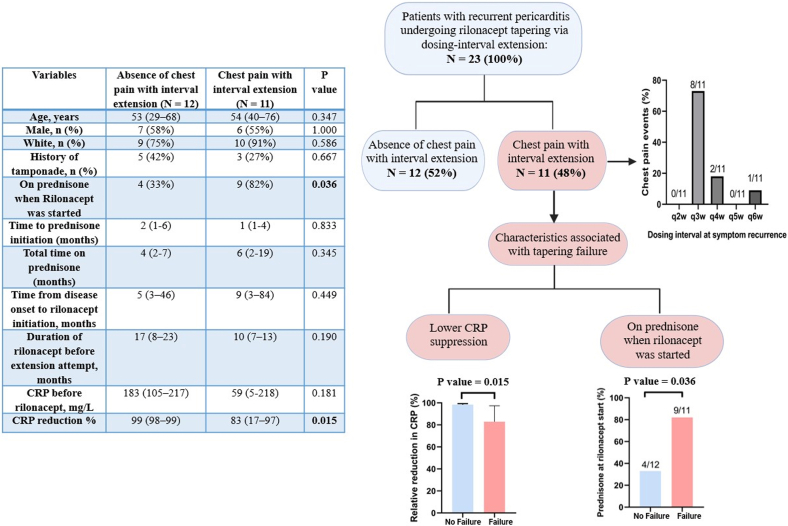
**Rilonacept Tapering via Dosing-Interval Extension in Recurrent Pericarditis: Chest Pain Outcomes and Factors Associated With Tapering Failure** Tapering cohort (n = 23) stratified by chest pain during interval extension (n = 11) vs no chest pain (n = 12). CRP = C-reactive protein.

Prednisone use at rilonacept initiation was significantly more frequent among patients who developed chest pain during tapering (9/11, 82%) compared with those who remained asymptomatic (4/12, 33%; OR: 9.0; 95% CI: 1.3-63.0; *P* = 0.036). Neither the timing of initiation nor the overall duration of prednisone therapy differed between groups. CRP levels before initiation of rilonacept were not different between groups, while the relative CRP reduction was significantly greater in patients tolerating interval extension (99% [98-99] vs 83% [17-97]; *P* = 0.015) (Figure 1). We performed a sensitivity analysis excluding the single patient with a CRP-positive flare during tapering to confirm that the between-group difference was not driven by this event; the difference in relative CRP reduction remained significant (99% [98-99] vs 90% [1-98]; *P* = 0.034).

## Discussion

Current guidelines endorse IL-1 inhibition with rilonacept for RP but provide limited guidance on treatment duration and tapering. Our experience offers a pragmatic view of rilonacept tapering in routine practice. We found that interval extension was feasible, but symptom recurrence was common and often required reverting to a shorter dosing interval, often driven by patient preference. Symptoms clustered during the transition from dosing every 2 weeks to every 3 weeks. Chest pain during interval extension was more frequently observed among patients receiving prednisone at rilonacept initiation, whereas greater on-treatment CRP suppression was associated with better tolerance of dose-spacing. Together, these observations may reflect residual, low-grade inflammation that is below the sensitivity of CRP or clinically quiescent under full IL-1 blockade but becomes symptomatic as dosing intervals lengthen. Prednisone use at initiation may likewise mark a more complex, prednisone-dependent phenotype with increased susceptibility to symptoms during tapering or an adverse effect of prednisone is causing recurrences.^4^ Rilonacept was well tolerated, with adverse events limited to mild injection-site reactions in 7 (30%) cases and no discontinuations due to serious toxicity. This stepwise protocol appeared safe, with no hospitalizations or worsening effusion requiring invasive procedures, and rapid response to rilonacept reinitiation when needed. These findings should be considered exploratory given the retrospective design, small sample size, lack of a control group, and nonstandardized tapering guided by physician judgment. Finally, because most taper-related events were symptom-based without biochemical relapse, outcome misclassification cannot be excluded.

## Conclusions

In this real-world experience with rilonacept tapering, interval extension was feasible but frequently limited by recurrent symptoms requiring a return to a shorter dosing interval. Prednisone use at rilonacept initiation and less on-treatment CRP suppression were associated with tapering failure, underscoring the need for prospective studies to compare tapering vs discontinuation strategies and to identify the patients most likely to tolerate treatment withdrawal.

## Funding support and author disclosures

The authors have reported that they have no relationships relevant to the contents of this paper to disclose.

## References

[1] Wang T.K.M., Klein A.L., Cremer P.C. (2025). 2025 concise clinical guidance: an ACC expert consensus statement on the diagnosis and management of pericarditis. J Am Coll Cardiol.

[2] Klein A.L., Imazio M., Cremer P. (2021). Phase 3 trial of Interleukin-1 trap rilonacept in recurrent pericarditis. N Engl J Med.

[3] Saraswati U., Bhalla J.S., Tereshchenko L.G. (2026). Comparative analysis of recurrence rates following various cessation strategies for rilonacept in recurrent pericarditis. Heart.

[4] Kelly J., Barbani Moglie I., Daly J. (2025). Prednisolone has a time-dependent shift in the effects on IL-1β release: from Anti- to pro-inflammatory. Circulation.

